# On the Structure and Properties of AlMgB_14_-TiB_2_ Composites Obtained from SHS Powders by Spark Plasma Sintering

**DOI:** 10.3390/ma14195521

**Published:** 2021-09-24

**Authors:** Pavel Nikitin, Ilya Zhukov, Aleksey Matveev, Sergei Sokolov, Mikhail Grigoriev, Alexander Vorozhtsov

**Affiliations:** Laboratory of Metallurgy Nanotechnologies, National Research Tomsk State University, Lenin Avenue, 36, 634050 Tomsk, Russia; gofra930@gmail.com (I.Z.); alekey.9595@mail.ru (A.M.); sokolovsd95@gmail.com (S.S.); mvgrigoriev@yandex.ru (M.G.); abv1953@mail.ru (A.V.)

**Keywords:** composites, AlMgB_14_, TiB_2_, SHS, spark plasma sintering, titanium diboride

## Abstract

AlMgB_14_-TiB_2_ composite materials were fabricated by self-propagating high-temperature synthesis (SHS) followed by spark plasma sintering of the obtained SHS products. It was found that, during the SHS, the AlMgB_14_ phase is formed at a donor (Ti + 2B) to acceptor (Al_12_Mg_17_-B) mass ratio of 3:7 and 4:6, respectively. The specimen sintered from the SHS powder with the donor:acceptor mass ratio of 5:5 at a temperature of 1470 °C has a uniform skeletal structure. The average hardness of the obtained specimen is 30.1 GPa.

## 1. Introduction

In 2000, B. Cook et al. reported promising properties of a ternary boride AlMgB_14_ [1]. First of all, it is a combination of high hardness and low friction coefficient. Since that moment, scientists from different countries have been actively investigating AlMgB_14_ materials and composite materials based on them: in particular, methods of fabricating bulk specimens [1,2,3,4], methods of obtaining coatings [5,6], formation [4,7] and decomposition [8] mechanisms, tribological characteristics of AlMgB_14_-based materials in dry conditions [7,9,10] and in various lubricants [11,12], and mechanical properties [1,2,3,4,7,8]. Over the past two decades, many methods have been reported for the production of AlMgB_14_-based materials, but these can be divided into two groups.

The first group includes methods for the production of bulk materials in situ (one-step method): the initial powders of aluminum, magnesium, and boron are simultaneously consolidated and sintered by hot pressing or spark plasma sintering (SPS). The main advantage of this method is the one-step process for obtaining AlMgB_14_. The disadvantage of this method is insufficient consolidation of the powder mixture due to the occurrence of secondary chemical reactions and the release of by-products. The second group includes methods for production of bulk materials by a two-step method [13,14]. In this case, at the first stage, the initial powders are sintered in a high-temperature vacuum furnace, and then the obtained prereacted AlMgB_14_ powder is consolidated (including with additives) by hot pressing or SPS. The advantage of the two-step method is the achievement of the greatest consolidation of the initial powders and the control of the phase composition in comparison with the one-step method.

It should be noted that two-stage methods are energy consuming. For example, in [3], the duration of AlMgB_14_ powder sintering in a high-temperature vacuum furnace was 3 h, which leads to an unjustifiably high energy consumption and is caused by the slow reaction behavior in the initial powder mixture. The above raises the question of the possibility of intensifying the reaction in an Al-Mg-B powder mixture, controlling the structure, and achieving homogeneity of AlMgB_14_ with various additives. In this regard, in our previous work [15], we proposed a method for obtaining AlMgB_14_-TiB_2_ composites using the technology of self-propagating high-temperature synthesis (SHS). This method is based on the exothermic reaction of titanium and boron with the formation of titanium diboride (donor), which is accompanied by the release of a large amount of heat. The (Ti + 2B) reaction is a heat source for the endothermic reaction of AlMgB_14_ formation in the Al_12_Mg_17_-B acceptor system. In our previous work, the donor:acceptor ((Ti + 2B):Al_12_Mg_17_-B) mass ratio was 3:7 [15]. The novelty of the proposed technology is as follows. To initiate a reaction in the SHS mode, it is necessary to spend several orders of magnitude less energy compared to high-temperature vacuum sintering, and the synthesis rate in the SHS mode is many times higher than the synthesis rate during vacuum sintering. In addition, using the SHS method, the phase composition and structure of the obtained products can be controlled in comparison with one-step methods [16]. In particular, in [17], the authors studied AlTi-TiB_2_ composite materials obtained by SHS from an Al-Ti-B powder mixture. It was found that the structure of the obtained materials is represented by titanium diboride particles, which are uniformly distributed in the AlTi matrix. The authors found that such a structure cannot be obtained by the traditional method of high-temperature vacuum sintering of Al and TiB_2_ powders. In [16], it was shown that the structure of the BN-TiB_2_ ceramic composite obtained from the (B + 0.5N_2_ + TiB_2_) system consists of a boron nitride matrix in which isolated particles of titanium diboride of various sizes are uniformly distributed. When the initial composition of the mixture changes to (Ti + 2B + BN), a BN-TiB_2_ composite of the same composition is formed; however, the structure of the SHS material changes to a skeletal structure.

Thus, of it is of particular interest to control the structure of AlMgB_14_-TiB_2_ composites obtained by the SHS method by changing the ratio of components (donor:acceptor) in the initial mixture, as well as to compare the obtained results with the results of other studies. The purpose of this work is to study the effect of varying the donor:acceptor ratio on the synthesis process, phase composition, structure, and properties of the obtained composite materials.

## 2. Materials and Methods

### 2.1. The First Step

To obtain AlMgB_14_-TiB_2_ composite mixture, powders of an Al_12_Mg_17_ intermetallic alloy (purity 98.8%, average particle size 0.6 μm) [7], amorphous boron (purity 98.8%, average particle size 0.6 μm), and titanium (purity 99.2%, average particle size 140 μm) were used as the raw precursors. To obtain the main mixture (acceptor), the Al_12_Mg_17_ intermetallic alloy and amorphous boron were mixed in an atomic ratio of 2:14. Powders of titanium and amorphous boron were mixed in a (69 wt% Ti + 31 wt% B) stoichiometric ratio to obtain a highly exothermic donor mixture (T_ad_ = 3193 K, 4250 kJ/kg). The stoichiometric ratio of the donor mixture was chosen to carry out experiments in a wide range of donor:acceptor ratios and to avoid an incomplete reaction with the formation of additional phases (TiB and B). The donor and acceptor systems were mixed in a mass ratio of 3:7, 4:6, 5:5, and 6:4.

The obtained powder mixtures were cold-pressed into 23 mm diameter samples. Obtained samples were placed in an SHS reactor, followed by the evacuation of the reactor and filling with argon. To ignite the samples, a current was applied to the molybdenum spiral in contact with the upper part of the samples. An intermediate (Ti + B) ignition layer was used for the uniform distribution of heat throughout the sample volume [15].

### 2.2. The Second Step

The obtained SHS samples were milled by hand in a mortar, placed in a 12.8 mm diameter graphite die, and then simultaneously sintered and consolidated by spark plasma sintering (DR. SINTER model SPS-625 Spark Plasma Sintering System was utilized). The pressure was 70 MPa and the sintering temperature varied from 1400 to 1500 °C at heating rates of 50 and 250 °C/min, respectively. The sintering temperature dependeds on the shrinkage process of the powder mixtures. The specimens were designated according to the donor:acceptor mass content (Table 1). After the completion of sintering, the SPSed samples were cooled in the switched-off mode. For further studies, the surface of the samples was polished with diamond pastes.

### 2.3. Characterization

The temperature of SHS synthesis was controlled using tungsten–rhenium thermocouples. The microstructures of the SHS powders and spark-plasma-sintered specimens were observed by a JEOL JSM-6490 microscope equipped with energy dispersive spectroscopy (EDX). The phase composition of the SHS powders and obtained specimens was identified using X-ray diffraction with CuKα radiation using a Shimadzu XRD 7000 diffractometer. The Diffrac.EVA program was used to determine the phase composition. The PDF-4 (powder diffraction file) database was used as a database. The densities of the specimens were calculated by the Archimedes method with distilled water as the immersion medium. A Metolab-502 tester was employed to measure the Vickers hardness. The dwell time was 10 s, and the normal load was 9.8 N. At least 10 indentations were made from different places of the samples.

## 3. Results and Discussion

### 3.1. SHS Powders

**Figure 1:**
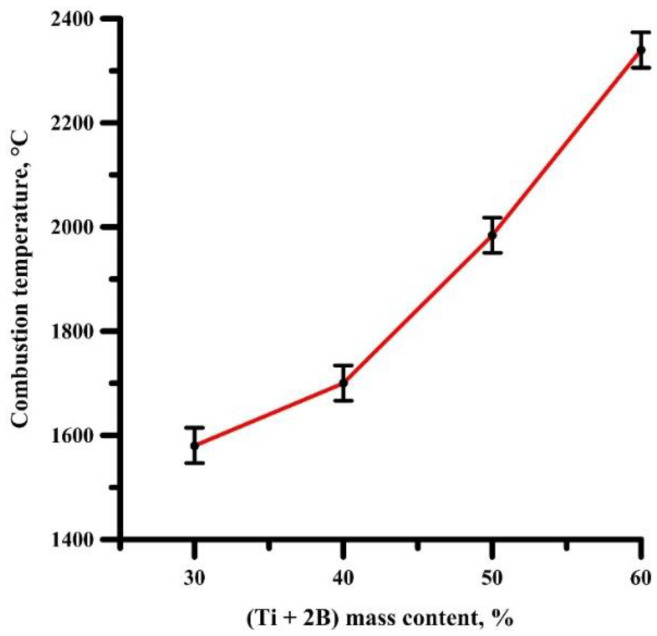
The dependence of the combustion temperature of the SHS powders on the donor mass content.

Figure 1 shows the dependence of the combustion temperature of composite powders in the SHS mode on the donor mass content. As can be seen from Figure 1, with an increase in the (Ti + 2B) donor content, the combustion temperature increases. The combustion temperatures of SHS powders with the donor:acceptor mass content of 3:7, 4:6, 5:5, and 6:4 are 1580, 1700, 1985, and 2340 °C, respectively. Thus, varying the donor:acceptor ratio in the initial mixture leads to a change in the combustion temperature of the mixtures, which makes it possible to control the synthesis processes. Presumably, a change in temperature leads to a change in the phase composition and structure of the SHS products.

**Figure 2:**
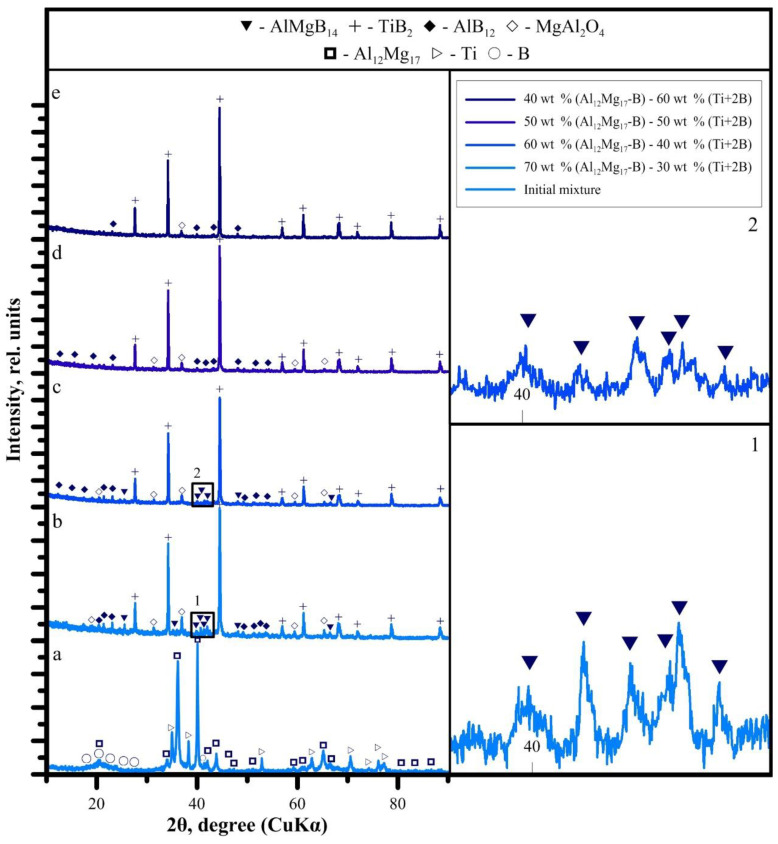
XRD patterns of the (**a**)—initial powder with the donor:acceptor mass ratio of 3:7; SHS powders with the donor:acceptor mass ratio of (**b**)—3:7, (**c**)—4:6, (**d**)—5:5, and (**e**)—6:4.

Indeed, a change in the combustion temperature leads to a change in the phase composition of SHS products. In accordance with XRD patterns of the obtained SHS powders (Figure 2), TiB_2_ peaks have the highest intensity. AlMgB_14_ peaks were found in SHS powders with the donor:acceptor mass content of 3:7 and 4:6. In other powders, the peaks in the 2θ = 40–43° region of the AlMgB_14_ phase are replaced by peaks of the AlB_12_ phase. Spinel MgAl_2_O_4_ was also found in all SHS powders.

**Figure 3:**
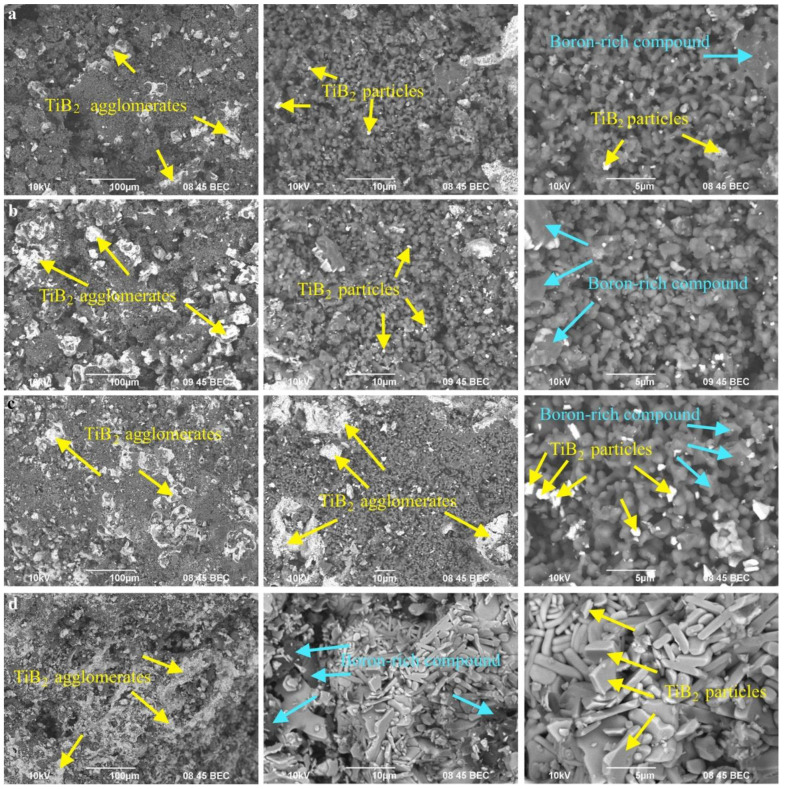
SEM image of the SHS powders with the donor:acceptor mass ratio of (**a**)—3:7, (**b**)—4:6, (**c**)—5:5, and (**d**)—6:4 (SE + BSE).

Figure 3 shows SEM images of the obtained SHS powders. SHS powders with (Ti + 2B) donor mass content of 30, 40, and 50 wt% are represented by particles and agglomerates (up to 30 μm in size) of titanium diboride distributed in a boron-rich matrix (apparently, AlMgB_14_/AlB_12_ phases). With an increase in the donor content, the size of the titanium diboride particles, as well as the concentration of their agglomerates, increases. The SHS powder with the donor mass content of 60 wt% is represented by agglomerates with an average size of 100 μm (Figure 3d), in which TiB_2_ particles have the form of elongated plates. In other words, varying the donor mass content changes the structure of the obtained SHS powders.

**Figure 4:**
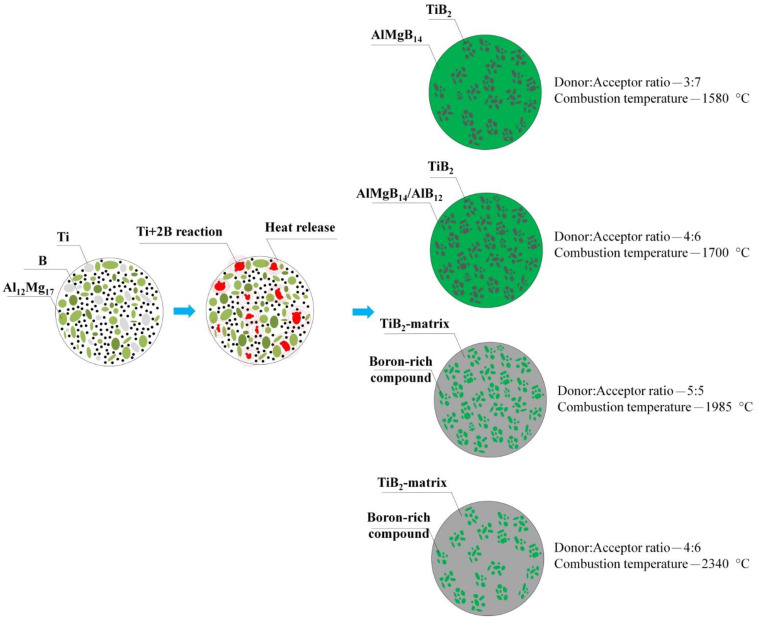
The mechanism of interaction between donor and acceptor in the SHS mode.

Thus, based on the obtained results, a mechanism for the interaction of the donor and acceptor was proposed, depending on their concentration in the initial mixture (Figure 4). During the combustion of the SHS powder with a donor:acceptor mass content of 3:7, the heat released from the exothermic reaction of the (Ti + 2B) donor is spent on heating the components of the acceptor mixture, followed by the synthesis reaction. With an increase in the donor concentration, the acceptor concentration decreases, and, as a consequence, the amount of heat required to initiate the reaction of AlMgB_14_ formation decreases. This leads to an increase in the combustion temperature of SHS powders. As the literature analysis shows, the optimum sintering temperature for AlMgB_14_ is 1400 °C [1,4,18]. In [8], the decomposition mechanism of AlMgB_14_ in the SPS process at temperatures above 1400 °C was investigated. It was found that local overheating of the initial powder mixture leads to the decomposition of AlMgB_14_ into Al_12_Mg_17_, AlB_12_, and Mg. In this work, in accordance with the obtained XRD patterns (Figure 2), the highest content of the AlMgB_14_ phase was achieved in the SHS powder with a minimum donor content of 30 wt% (combustion temperature—1580 °C). An increase in the (Ti + 2B) donor content leads to a decrease in the AlMgB_14_ content (40 wt% of donor) and, ultimately, to the decomposition of AlMgB_14_ into AlB_12_ and Mg, which evaporates due to the low boiling point (50 and 60 wt% of donor). Thus, during thermochemical-coupled self-propagating high-temperature synthesis, local overheating of the powder mixture is observed. In addition, with an increase in the donor concentration, more titanium diboride particles are formed in the synthesis products, and the interface (acceptor products) between these particles decreases. This leads to recrystallization, sintering, and the formation of agglomerates of titanium diboride particles. At a combustion temperature of 2340 °C (donor:acceptor ratio of 6:4), an intensive growth of TiB_2_ particles (they form agglomerates up to 100 μm in size) and a change in their shape to elongated plates are observed.

Thus, a change in the donor concentration in the initial mixture leads to a change in the synthesis temperature, phase composition, and structure of the SHS products: in particular, the shape and size of titanium diboride particles. It is assumed that such a change in the structure of SHS powders will lead to a change in the structure of composites obtained by spark plasma sintering of these SHS powders.

### 3.2. Spark Plasma Sintered Specimens

**Figure 5:**
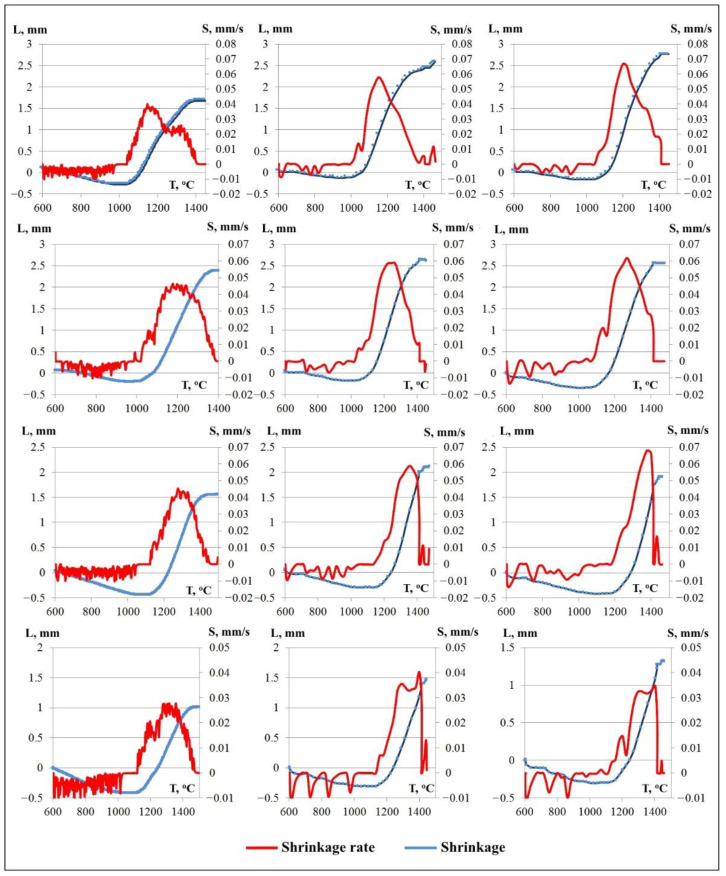
Typical shrinkage curves for the obtained specimens.

Spark plasma sintering conditions are shown in Table 2. Typical shrinkage curves for specimens are shown in Figure 5. At a heating rate of 50 °C/min, specimen #30-1 shrinks intensively within the temperature range from 1040 to 1410 °C. Two peaks of shrinkage at T = 1150 °C (S = 0.04 mm/s) and T = 1340 °C (S = 0.025 mm/s) were found. An increase in the heating rate to 250 °C/min leads to the merging of the shrinkage peaks, a decrease in the temperature range of shrinkage, and an increase in the maximum shrinkage rate to 0.55–0.65 mm/s (specimens #30-2, #30-3). Isothermal holding leads to an increase in the shrinkage (the specimen #30-3).

At the heating rate of 50 °C/min, specimen #40-1 shrinks intensively within the temperature range from 1020 to 1400 °C. The maximum shrinkage rate (S) of 0.04 mm/s is observed at a temperature of 1200 °C, and a slightly delineated peak of the shrinkage rate is also observed at a temperature of 1080 °C (S = 0.017 mm/s). An increase in the heating rate to 250 °C/min leads to a shift in the temperature range of intense shrinkage to 1020–1460 °C, a temperature shift corresponding to the maximum shrinkage to 1260 °C, and an increase in the maximum shrinkage rate (S) to 0.06 mm/s (specimens #40-2, #40-3). Isothermal holding leads to an increase in the shrinkage (the specimen #40-3).

At the heating rate of 50 °C/min, specimen #50-1 shrinks intensively within the temperature range from 1120 to 1500 °C. The maximum shrinkage rate (S) of 0.04 mm/s is related to a temperature of 1320 °C (specimen #50-1). An increase in the temperature rate to 250 °C/min leads to a shift in the temperature range of intense shrinkage to 1020–1470 °C, a temperature shift corresponding to the maximum shrinkage to 1380 °C, and an increase in the maximum shrinkage rate to 0.06 mm/s (specimens #50-2, #50-3). Isothermal exposure leads to a slight decrease in shrinkage (the specimen #50-3).

At the heating rate of 50 °C/min, specimen #60-1 shrinks intensively within the temperature range from 1110 to 1510 °C. Two peaks of shrinkage at T = 1200 °C (S = 0.018 mm/s) and T = 1330 °C (S = 0.026 mm/s) were found. An increase in the temperature rate to 250 °C/min (specimens #60-2, #60-3) leads to a shift in the temperature range of intense shrinkage to 1110–1470 °C and the appearance of a third peak on the temperature dependence of the shrinkage rate: T = 1200 °C (S = 0.007–0.014 mm/s), T = 1320 °C (S = 0.03 mm/s), and T = 1400 °C (S = 0.04 mm/s). Isothermal exposure leads to a slight decrease in shrinkage (the specimen #60-3).

**Figure 6:**
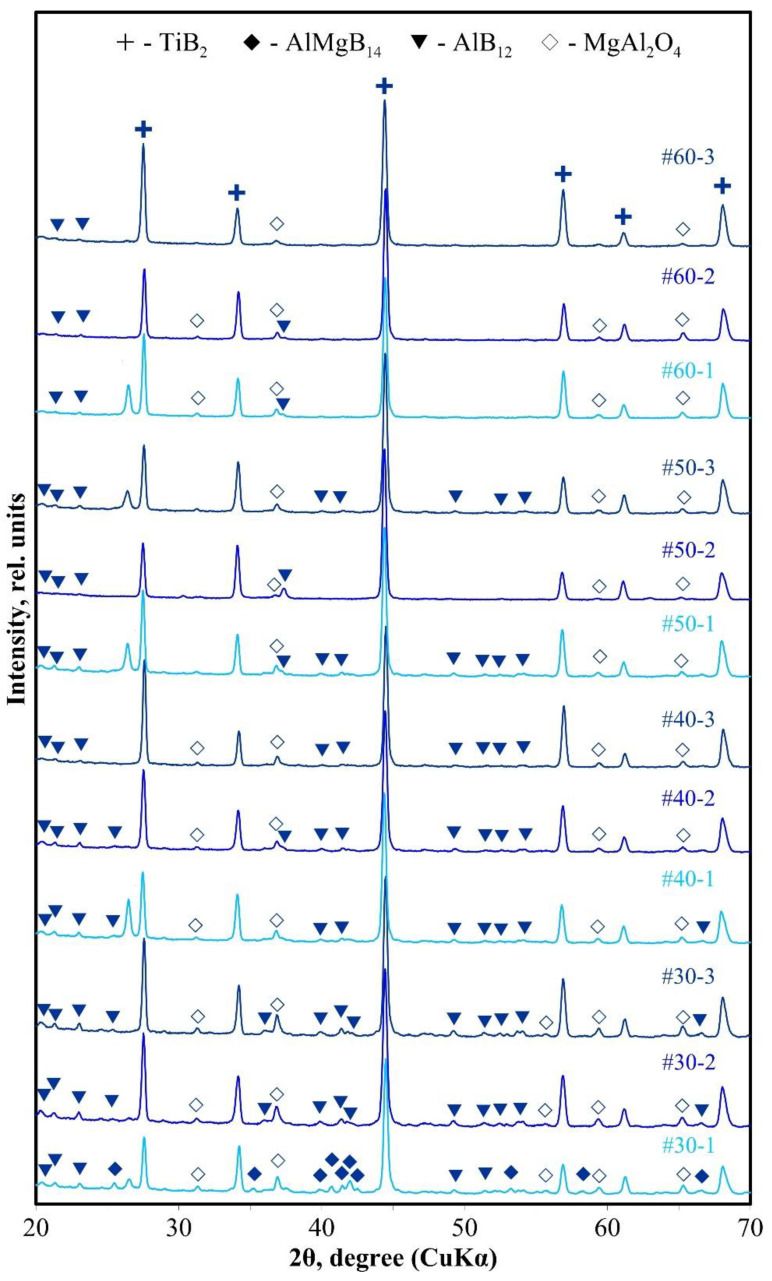
XRD patterns of the SPSed specimens.

As can be seen from XRD patterns of the sintered specimens (Figure 6), the highest intensity is observed for titanium diboride peaks. AlMgB_14_ was found only in the XRD pattern of the specimen #30-1. This specimen also contains AlB_12_. With an increase in the sintering temperature (specimens #30-2, #30-3), the AlMgB_14_ peaks are replaced by the AlB_12_ peaks, and an increase in the intensity of the titanium diboride peaks is also observed. The AlMgB_14_ phase was not found in other specimens. Apparently, this can be due to two reasons. First, during the SHS synthesis, the AlMgB_14_ phase is decomposed (Figure 2). Second, an analysis of the XRD patterns given in other works [9,19] shows that even in AlMgB_14_-TiB_2_ composites with a titanium diboride content of 30 wt%, the height of the titanium diboride peaks on XRD-patterns significantly exceeds the height of the AlMgB_14_ peaks, smoothing them, as in this work. In other words, the crystal lattice of titanium diboride reflects electrons more intensely than the crystal lattice of AlMgB_14_. In this case, in specimens with a donor content in the SHS mixture of 50 and 60 wt% peaks in the 2θ = 40–43° of the AlB_12_ phase are smoothed out. Spinel MgAl_2_O_4_ was also found in all specimens [20,21].

**Figure 7:**
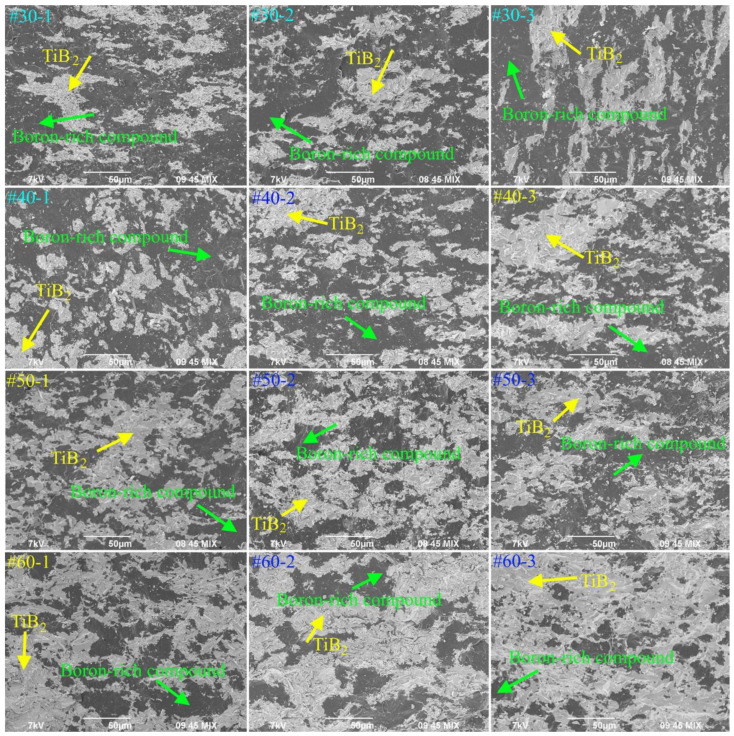
SEM images of the obtained specimens (SE + BSE).

SEM images of the fracture surface of the obtained specimens are shown in Figure 7. The formation of light-colored agglomerates of titanium diboride is associated with the presence of agglomerates in the SHS powders. The fracture surface of specimens with a donor content of 30 and 40 wt% is represented by islands of titanium diboride agglomerates (light areas) distributed in a boron-rich matrix (dark areas) of AlMgB_14_/AlB_12_. On the contrary, on the fracture surfaces of specimens with a donor content of 50 and 60 wt% large agglomerates of titanium diboride form a skeletal structure, in which boron-enriched compounds (most likely, AlB_12_) are embedded. This is consistent with the XRD results. Thus, the structure of sintered specimens inherits the structure of SHS powders.

**Figure 8:**
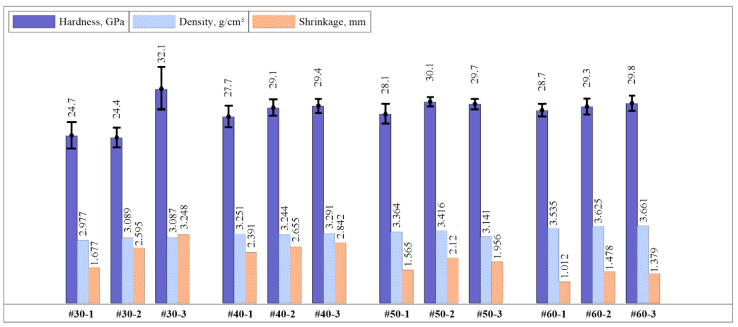
Histograms of the distribution of the hardness value, density, and shrinkage of the specimens.

The properties of the obtained specimens are shown in Table 3. Histograms of the distribution of the hardness value, density, and shrinkage of the specimens sintered under different modes are shown in Figure 8. It was found that in the specimens obtained from the SHS powder with a donor content of 30 wt% (#30-1, 30-2, 30-3), with an increase in the heating rate (isothermal holding of 5 min), the greatest shrinkage, density, and, as a consequence, the highest hardness of 31.9 ± 3.5 GPa are achieved (the specimen #30-3). However, in these specimens, there is a large deviation in hardness values depending on the indentation region. This is due to the non-uniform structure of the obtained composites.

In specimens obtained from the SHS powder with a donor content of 40 wt% (#40-1, 40-2, 40-3), with an increase in the heating rate and the use of isothermal holding, shrinkage, density, and hardness linearly increase. The specimen #40-3 has the highest hardness of 29.4 ± 1.2 GPa.

In specimens obtained from the SHS powder with a donor content of 60 wt% (#60-1, 60-2, 60-3), with an increase in the heating rate and the use of isothermal holding, shrinkage, density, and hardness linearly increase, as in specimens with a donor content of 40 wt%. The specimen #60-3 has the highest hardness of 29.8 ± 1.3 GPa.

In specimens obtained from the SHS powder with a donor content of 50 wt% (#50-1, 50-2, 50-3), the highest average hardness of 30.1 GPa has the specimen sintered at a temperature of 1470 °C with a heating rate of 250 °C/min without using isothermal holding (#50-2). In this case, the hardness values in different areas of the material (Table 4) have the minimum deviation from the average hardness value and, as a consequence, the smallest error of 0.8 GPa among all specimens.

**Figure 9:**
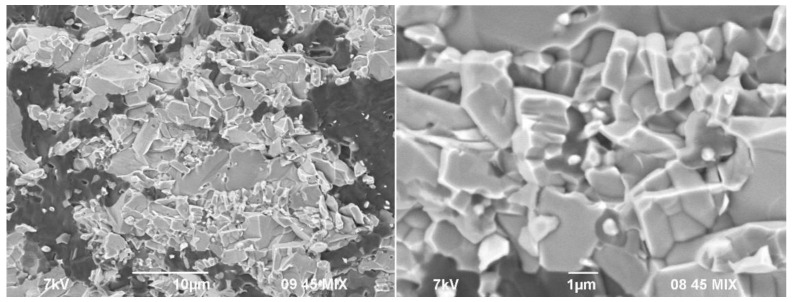
Fracture surface of the specimen #50-2 (SE + BSE).

Probably, the obtained material has the most uniform structure in comparison with the other specimens. Indeed, when comparing SEM images of SHS powders (Figure 3) and spark-plasma-sintered specimens (Figure 6), it was found that during spark plasma sintering of SHS powders, recrystallization of titanium diboride particles and agglomerates occurs, which form island (specimens #30-1, 30-2, 30-3; #40-1, 40-2, 40-3) and skeletal (#50-1, 50-2, 50-3; #60-1, 60-2, 60-3) structures. Thus, under the selected conditions, sintering of the SHS powder with the (Ti + 2B) donor mass content of 50 wt% is optimal for obtaining composite materials with a uniform skeletal structure, represented by TiB_2_ agglomerates containing particles with 1 μm in size (Figure 9).

Thus, varying the donor:acceptor ratio in the initial powder mixture makes it possible to control the structure, phase composition, and properties of spark-plasma-sintered AlMgB_14_-TiB_2_ composites. In particular, this makes it possible to achieve a unique skeletal structure of the composite #50-2 with a uniform distribution of phases and, as a consequence, to achieve a minimum deviation in hardness values (0.8 GPa) in comparison with the composite obtained in our previous work (3.5 GPa, specimen #30-3) [15]. It should be noted that such a structure cannot be obtained by classical sintering of AlMgB_14_ and TiB_2_ powders, which is an important advantage over traditional methods for preparing AlMgB_14_-TiB_2_. At the same time, the properties of the obtained composites are in good agreement with the properties of the composites obtained in other works (Table 5).

## 4. Conclusions

In summary, the fundamental possibility of controlling the structure, phase composition, and properties of AlMgB_14_-TiB_2_ composite materials obtained by SHS with subsequent spark plasma sintering by varying the donor:acceptor ratio in the initial powder mixture was investigated. During the SHS, AlMgB_14_ is formed at the donor:acceptor mass ratio of 3:7 and 4:6. The specimen sintered from the SHS powder with the donor:acceptor mass ratio of 5:5 at a temperature of 1470 °C has a uniform skeletal structure and an average hardness value of 30.1 GPa.

## Figures and Tables

**Table 1:** Specimen designations depending on the donor:acceptor ratio.

Specimen	#30-1	#30-2	#30-3	#40-1	#40-2	#40-3	#50-1	#50-2	#50-3	#60-1	#60-2	#60-3
donor:acceptor mass ratio	3:7	3:7	3:7	4:6	4:6	4:6	5:5	5:5	5:5	6:4	6:4	6:4

**Table 2:** Spark plasma sintering conditions.

Specimen	Temperature, °C	Heating Rate, °C/min	Isothermal Holding, min	Pressure, MPa
#30-1	1450	50	0	70
#30-2	1470	250	0	70
#30-3	1470	250	5	70
#40-1	1400	50	0	70
#40-2	1470	250	0	70
#40-3	1470	250	5	70
#50-1	1500	50	0	70
#50-2	1470	250	0	70
#50-3	1470	250	5	70
#60-1	1510	50	0	70
#60-2	1470	250	0	70
#60-3	1470	250	5	70

**Table 3:** Properties of the obtained specimens.

Specimen	Density, g/cm^3^	* Theoretical Density, g/cm^3^	Hardness, GPa
#30-1	2.977	2.97	24.7 ± 2.2
#30-2	3.089		24.4 ± 1.6
#30-3	3.087		32.1 ± 3.5
#40-1	3.251	3.12	27.7 ± 1.8
#40-2	3.244		29.1 ± 1.4
#40-3	3.291		29.4 ± 1.2
#50-1	3.364	3.29	28.1 ± 1.6
#50-2	3.416		30.1 ± 0.8
#50-3	3.141		29.7 ± 0.9
#60-1	3.535	3.48	28.7 ± 1.1
#60-2	3.625		29.3 ± 1.3
#60-3	3.661		29.8 ± 1.3

*—Theoretical density of the X wt% AlMgB_14_—Y wt% TiB_2_

**Table 4:** Hardness values of the specimen #50-2.

Hardness, GPa	29.2	30.1	30.7	31.7	30.1	29.6	32.1	29.3	29.2	29.2	29.2	30.1
D1, µm	24.88	24.42	22.10	24.12	24.09	25.12	23.34	25.15	25.30	24.63	24.88	24.42
D2, µm	25.54	25.18	24.21	24.24	25.57	24.91	24.72	25.15	25.06	25.75	25.54	25.18

**Table 5:** Comparison of hardness values with other studies.

Material	Hardness, GPa	Reference
#50-2	30.1 ± 0.8	This work
#30-1	32.1 ± 3.5	This work
AlMgB_14_ + 30 wt% TiB_2_	27.67 ± 0.6	[2]
AlMgB_14_ + 50 wt% TiB_2_	27.92 ± 0.82	[2]
AlMgB_14_ + 30 wt% TiB_2_	32.5 *	[9]

*—The deviation was not reported

## Data Availability

The data presented in this study are available in the article.
